# Emerging Wireless Sensor Networks and Internet of Things Technologies—Foundations of Smart Healthcare

**DOI:** 10.3390/s20133619

**Published:** 2020-06-27

**Authors:** Gordana Gardašević, Konstantinos Katzis, Dragana Bajić, Lazar Berbakov

**Affiliations:** 1 Faculty of Electrical Engineering, University of Banja Luka, 78000 Banja Luka, Bosnia and Herzegovina; gordana.gardasevic@etf.unibl.org; 2 Department of Computer Science and Engineering, European University Cyprus, 2404 Nicosia, Cyprus; k.katzis@euc.ac.cy; 3 Faculty of Technical Science, University of Novi Sad, 21000 Novi Sad, Serbia; draganab@uns.ac.rs; 4 Institute Mihajlo Pupin, University of Belgrade, 11060 Belgrade, Serbia

**Keywords:** Internet of Things, Internet of Medical Things, smart healthcare, wireless sensor networks, communication technologies and protocols, crowdsourcing/crowdsensing

## Abstract

Future smart healthcare systems—often referred to as Internet of Medical Things (IoMT) – will combine a plethora of wireless devices and applications that use wireless communication technologies to enable the exchange of healthcare data. Smart healthcare requires sufficient bandwidth, reliable and secure communication links, energy-efficient operations, and Quality of Service (QoS) support. The integration of Internet of Things (IoT) solutions into healthcare systems can significantly increase intelligence, flexibility, and interoperability. This work provides an extensive survey on emerging IoT communication standards and technologies suitable for smart healthcare applications. A particular emphasis has been given to low-power wireless technologies as a key enabler for energy-efficient IoT-based healthcare systems. Major challenges in privacy and security are also discussed. A particular attention is devoted to crowdsourcing/crowdsensing, envisaged as tools for the rapid collection of massive quantities of medical data. Finally, open research challenges and future perspectives of IoMT are presented.

## 1. Introduction

Improved living conditions and availability of quality healthcare have increased the life expectancy and aging population share in most of the countries. In the report on World Population Prospects: the 2019 Revision [1], it was stated that in 2018, for the first time in history, persons older than 65 years have outnumbered children under the age of 5. Moreover, the projections in the world population indicate that by 2050 the population above the age of 65 will be twice the size of population under the age of 5. At the global level, by 2050, 16% of population will be aged above 65 years, in contrast to 9% in the year 2019. These trends are expected to result in an increased incidence rate of chronic diseases and disabilities which will require healthcare systems to adopt new approaches in organization and advancements of the medical treatments [2].

Recent advancements in wireless communications and microelectronics, in addition to subsequent price reductions, have enabled the utilization of Wireless Sensor Networks (WSNs) in a myriad of innovative applications such as smart homes, military surveillance, intelligent traffic control, environmental monitoring, and so forth. WSNs are widely used in healthcare applications due to their advantages and diversity. Intensified efforts in providing advanced Internet of Things (IoT) healthcare architectures have stimulated innovative applications and have provided tremendous progress in this field. Smart healthcare is a promising WSN IoT application area, that will undoubtedly bring significant benefits to citizens. In particular, such applications will remotely deliver healthcare services, thus bringing significant cost savings as well as allowing patients to live independently in their own homes or in a family environment for longer periods of time. These systems will also enable early detection and preemption of diseases and significant improvement of patients’ quality of life. Novel smart healthcare architectures will benefit from deep learning, big data, and high-performance computing to implement reliable Cloud/Edge/Fog network solutions [3].

The design and deployment of such systems is not a trivial task. Therefore, open research problems in the area of WSNs, which represent one of the main parts of future IoT systems, have attracted the attention of many research groups. In particular, different aspects of energy-efficient communication, coding, collaborative techniques, privacy and security mechanisms, and other relevant topics have sparked the interest of the research community. Smart healthcare systems rely on different technologies: sensors and actuators for data acquisition and control actions, communication modules for data transmission, a real-time database for data storage, and finally, applications for data processing and visualization. All the aforementioned components have to be selected and integrated having in mind different constraints such as cost, comfort, reliability, and usability. The selection of a particular communication technology for healthcare applications strongly depends on the characteristics of the IoT environment, the number of sensor devices, energy requirements, network scalability, and so forth. Therefore, in this paper we have discussed and analyzed various low-power IoT communication technologies suitable for smart healthcare applications.

The organization of the paper is as follows. Section 2 presents an overview of emerging IoT communication technologies and standards, where the particular emphasis has been given to low-power wireless technologies at different scales. To address requirements and challenges of wireless communications for smart healthcare, a study of the physical (PHY) and Media Access Control (MAC) characteristics has been provided. IEEE 802.15.4 standard and its derivations such as IPv6 over Low - Power Wireless Personal Area Networks (6LoWPAN) and IPv6 over Time Slotted Channel Hopping (6TiSCH) are discussed in Section 2.1. Bluetooth and Bluetooth Low Energy (BLE) as one of the key enablers for smart healthcare are also addressed. Long-range low-power wireless IoT solutions enabled by LoRaWAN, SigFox, INGENU and WEIGHTLESS are presented in Section 2.2. This section also presents low-power long-range technologies that have been designed to operate on licensed bands. Section 2.3 addresses challenges and opportunities in security and privacy for smart healthcare. Emerging trends and applications for smart healthcare are discussed in Section 3. Section 3.1 is devoted to the application domains including patients, doctors, and hospitals, as well as to the general architecture of the IoT healthcare system. Data harvesting in terms of mobile crowdsensing is described in Section 3.2, outlining several applications. Section 4 is devoted to the technical challenges in smart healthcare. Connected medical devices should be designed and implemented in such a way to prevent the risk of the patient’s safety. Finally, a conclusion highlights key findings of this survey by indicating open issues and future trends in the emerging field of smart healthcare.

### Contributions

In this paper, we present and discuss the main aspects and challenges in providing the communication framework for IoT smart healthcare. In particular, the main contributions of this paper can be summarized as follows:

- An extensive survey on emerging communication standards and technologies suitable for smart healthcare applications is presented. A particular emphasis has been given to the latest IoT standards and technologies. The specific requirements in terms of data rates, latency, and energy efficiency are discussed.
- An overview of security and privacy issues, as the major challenge for future smart healthcare systems, is presented.
- Emerging trends and applications for healthcare are presented. Particular attention is devoted to crowdsourcing/crowdsensing, envisaged as tools for the rapid collection of massive quantities of medical data.
- Finally, open research and technical challenges in designing an IoT system for smart healthcare are discussed.

## 2. Communication Technologies for WSN-Based Healthcare

### 2.1. LR-WPAN Standards and Technologies

WSNs are one of the key IoT technologies with applications in numerous fields such as infrastructure surveillance, home and industrial automation, smart cities, intelligent transportation systems, healthcare systems, and so forth. An efficient WSN-based IoT healthcare system enables real-time remote monitoring of patient status, contextual and environmental information, prevention of critical conditions, quality of life improvements, administration of medical equipment and drug databases, and so forth. IoT sensor devices in healthcare applications should provide reliable measurement, control, and analysis of vital health indicators, such as the electrocardiogram (ECG), blood pressure, blood glucose level, oxygen saturation, and so forth [4,5,6,7]. This data is then aggregated, and features are extracted and transmitted to the Cloud or Fog for further examination or use by machine intelligence algorithms.

Data acquisition is the first component of an IoT healthcare system, which gathers physiological and ambient signals, primarily through Wireless Body Area Networks (WBANs) sensors but also sensors deployed in the environment. WBANs represent one of the core IoT healthcare technologies, where the development of reliable sensors is essential for the proper operation of a complete system [8]. IEEE 802.15.6 is a reference standard for WBANs and it aims to provide low-power, short-range, and extremely reliable wireless communication outside and inside of the human body. The current IEEE 802.15.6 standard defines three PHY layers, that is, Narrowband (NB), Ultra-wideband (UWB), and Human Body Communications (HBC) layers depending on the application. The Medium Access Control (MAC) layer provides Carrier-Sense Multiple Access with Collision Avoidance (CSMA/CA), Time Division Multiple Access (TDMA), as well as the unscheduled access [9]. IEEE 802.15.6 categorizes WBAN applications into medical and non-medical, which may vary from military, sports, entertainment to healthcare. According to Reference [10], medical applications of IEEE 802.15.6 are divided into three categories. The first one is the Wearable WBAN, the second is the Implant WBAN and the third is the remote control of medical devices. The main requirements of the standard have been defined so that the bit rates are in the range of 10 kbps to 10 Mbps. The nodes should easily be removed and added under 3 seconds and they should support mobility. Latency below 125 ms is expected for medical applications and less than 250 ms for non-medical applications. In-body and on-body nodes should be able to coexist while the Packet Error Rate (PER) should ideally be below 10% for a 256 octet payload for a majority (95%) of the best-performing links.

A variety of WBAN and WPAN protocols have been proposed in the literature to address diverse applications and scenarios. However, ZigBee, Bluetooth Low Energy (BLE), and Wi-Fi have received the widest adoption. Short-range communication technologies such as BLE [11], Radio Frequency Identification (RFID) [12], Near Field Communication (NFC) [13], ZigBee [14,15], IPv6 over Time-Slotted Channel Hopping - TSCH mode of IEEE 802.15.4e (6TiSCH) [16] are commonly used to transmit sensor data to a central entity that aggregates and processes the received data. Recently, IEEE 802.11ah, marketed as Wi-Fi HaLow, appeared as the amendment of the IEEE 802.11-2007 wireless networking standard, particularly adapted for better coverage and power-efficient IoT and M2M applications [17]. Long-range technologies, such as Low Power - Wide Area Network (LP-WAN) [18] or Long Term Evolution (LTE) [19], provide the transfer of the processed data to the patient or doctor premises for further examination and analysis.

WSNs’ reference standard for Low Rate - Wireless Personal Area Networks (LR-WPAN) is the IEEE 802.15.4 that defines modes on PHY and Data Link Layer (DLL) layers of the protocol stack, and is designed for short-range and low-power communications [20]. ZigBee is one of the most popular solutions, especially in the field of home automation and smart city applications. The typical transmission range that can be achieved in these networks is 10 to 100 m, with transmit power levels between 0 dBm and 20 dBm, respectively. Data rates vary from 20 kbps (868 MHz band) to 250 kbps (2.4 GHz band). Since these are loss-prone networks, with low-power levels, the term Low-power and Lossy Networks (LLN) is also used in the literature [21]. Above IEEE 802.15.4 PHY and MAC layers, standardized higher layer protocols can be implemented, such as for example, IPv6 over Low - Power Wireless Personal Area Networks (6LoWPAN) [22], Routing Protocol for Low-Power and Lossy Networks (RPL) [23], Constrained Application Protocol (CoAP) [15], and so forth.

IEEE 802.15.4-2003 defines three variants of the PHY layer, with applications in the 868 MHz, 915 MHz, and 2.4 GHz bands, and using one, ten, and sixteen channels, respectively. The channels are numbered from 11 to 26, where the distance between the two adjacent channels is 5 MHz. The problem that appears in Industrial, Scientific and Medical (ISM) frequency bands is the coexistence with other technologies, as the interference often occurs. In addition to the reference ZigBee standard, other standards that allow mesh networking in LR-WPAN are: WirelessHART, ISA SP100.11a, Z-Wave, WIA-PA, ANT, IP500, Wi-SUN, and so forth.

Traditional IEEE 802.15.4 WSN solutions have not considered the reliability of transmitted packets in a multihop topology. The limitations of the IEEE 802.15.4 MAC layer in mesh-networking mainly appear due to the use of a single channel and high power consumption as a result of the permanently active relay nodes. ZigBee does not provide high reliability and robustness since it does not employ protection against interference and fading effects. There have also been a considerable number of studies that tackled the problem of coexistence from the perspective of the 802.15.4 PHY layer, as ZigBee operates in the same frequency band as Wi-Fi and Bluetooth [24]. ZigBee 3.0 is the most recent version, which has been optimized for Machine to Machine (M2M) applications, and for low duty cycle and low latency operations [25]. However, ZigBee-based solutions need a significant improvement in order to become suitable for emergency information messages and vital sign monitoring in healthcare applications.

Bluetooth Low Energy (BLE), also known as Bluetooth Smart, was first introduced in 2006 as “Wibree” and in 2010 merged into the main Bluetooth standard with the adoption of the Bluetooth Core Specification Version 4.0 [26]. BLE has emerged as a popular low-power wireless technology for wireless sensing and the IoT, as many portable devices such as smartphones, laptops, and smartwatches are equipped with embedded BLE modules. Unlike classic Bluetooth optimized for continuous data streaming, BLE is optimized for short burst packet transmissions. Bluetooth 5.0 provides customized software optimized for medical applications [27]. This specification introduces enhancements in terms of higher data rate, interoperability, compatibility, and coverage by using increased transmit power and coded physical layer. Bluetooth 5.1, the latest version of Bluetooth specifications, provides support for high accuracy and interoperable positioning systems.

In smart healthcare applications, a small delay in data transmission is one of the basic requirements for time-critical operations. Therefore, it is necessary to introduce traffic prioritization and Quality of Service (QoS) mechanisms for the above-mentioned technologies [28]. Moreover, collisions that may occur while using communication technologies can cause data loss and potentially life-threatening situations. Therefore, it is essential to develop robust protocols to ensure safe and optimal operational conditions for medical devices. In order to reduce the negative effects of interference and multipath fading and to provide better reliability, efficient transmission mechanisms have been developed within new standard amendments: IEEE 802.15.4e, IEEE 802.15.4m, IEEE 802.15.4g, and others. Table 1 summarizes the main operational parameters of low-power wireless technologies suitable for smart healthcare applications.

The development of wireless sensor devices has evolved from simple tags, with applications in identification, tracking of goods, services, and so forth, to complex devices with advanced storage and information processing capabilities. The functionality of next-generation sensor devices, such as self-organizing and cooperative data processing, greatly enhances the performance of traditional WSNs, especially in specific applications, such as for example, industrial and healthcare IoT applications. In the last 20 years, smart sensor technologies have been the subject of intensive research activities, intending to adopt the necessary standards to support the “plug-and-play” concept, in which sensors from different manufacturers will be compatible and their replacement will be realized in a simple and efficient way [29]. Thanks to the advanced features of the integrated circuit design and packaging process, as well as an affordable cost of implementation, modern electronic systems have become an integral part of IoT sensor networks. Advances in the development of semiconductor technologies have made it possible to increase the processing and memory capacities of sensor devices. Solutions offered by the accelerated development of Micro Electro-Mechanical Systems (MEMS) are expected to provide even better performance and versatility of sensor platforms. There are already numerous variants of miniature MEMS sensors on the market that are used to measure different physical, chemical, and other types of signals [30].

#### Advances in LR-WPAN Networking

Demanding functionalities of the next-generation sensor devices also impose higher energy consumption, that is, the need to replace the battery more frequently, which affects network operations and increases the implementation cost. Generally, MAC mechanisms in WSNs are based on duty-cycling as a traditional approach for energy savings. Numerous algorithms have been proposed to adapt the MAC parameters of LR-WPANs to a broad range of applications [31,32]. However, energy efficiency and communication reliability are often conflicting requirements. Novel solutions such as Wake-up Radio (WuR) allow nodes to work at a power consumption level that is 1000 times lower than that of the traditional radio. Data communication among nodes is triggered in an on-demand manner, by either a sender or a receiver, which provides a significant reduction of energy consumption and improves the transmission reliability and packet delay [33,34].

New transmission mechanisms within the MAC sublayer, such as for example, IEEE 802.15.4e - TSCH provides an ultra-low duty cycle less than 0.1%, thus extending battery life by up to 10 years [35]. TSCH combines time synchronization with channel hopping to provide deterministic delay guarantees, communication reliability (i.e., resistance to interference and multipath fading), and higher network throughput in comparison to other MAC protocols. IEEE 802.15.4e represents the so-called “MAC amendment” and replaces the implementation of the IEEE 802.15.4 MAC sublayer without modifying the existing PHY. In this way, IEEE 802.15.4e can be viewed as a “software update” that works just like any other IEEE 802.15.4 radio chip. All nodes in the TSCH network are globally synchronized, thus providing 99.999% transmission reliability.

The TSCH communication uses 16 different channels denoted as channelOffset that is, an integer value from the range [0, .., 15]. TSCH creates the scheduling matrix based on [timeslot, channeloffset] pairs assigned to each communication link in the network. Typically, each timeslot is 10 ms long and supports the transmission/reception of a single packet along with its acknowledgment. By using this technique, TSCH provides guarantees for accessing the transmission medium, which is particularly important for real-time healthcare applications.

The scheduling in 6TiSCH networks has attracted considerable research interest. The 6TiSCH architecture defines several approaches for resource allocation: static scheduling, centralized monitoring and schedule management, “neighbor-to-neighbor” and “hop-by-hop” scheduling. The main goal in the design of the 6TiSCH network is to support different types of traffic and applications, based on a special module (6top) in the communication stack that will provide allocation policies. Current 6TiSCH implementations use the 2.4 GHz band, with 16 frequencies available. Privacy issues are particularly important in IoT healthcare applications. The relevant step in 6TiSCH convergence is the implementation of control plane protocols in order to assign required resources to the scheduling, routing, and application needs. The results obtained by using the 6TiSCH technology for smart healthcare are promising. The authors of Reference [36] present SPHERE (Sensor Platform for Healthcare in a Residential Environment) TSCH-based network stack for data collection in smart homes for healthcare. Trials performed in 29 public deployments show that this system provides required performance in terms of reliability (99.96% average PDR), schedule capacity (with up to 22 pps average packet rate), and energy efficiency (less than 9mAh consumption per day on environmental sensors).

The 6TiSCH has also defined secure light-weight join processes combining link-layer security features with a secure joining procedure using CoAP [37]. All these characteristics, particularly high reliability, deterministic latency, and ultra-low operations, make a 6TiSCH suitable for numerous healthcare applications. In order to enable wider adoption of 6TiSCH technology in various IoT scenarios and commercial applications, it is necessary to create a 6TiSCH benchmark platform that enables reliable and certified performance testing. 6TiSCH Open Data Action (SODA) is a research project that aims to provide the aforementioned testing conditions in accordance with standards and technical specifications, as well as to automate the measurement process [38]. The characteristics of individual applications (smart city, healthcare, industrial automation) are "mapped" into the appropriate parameters of the testbed or simulator. This requires a precise definition of transmitting powers, the number of hops, scheduling and routing protocols, and so forth. The final performance is assessed based on standard Key Performance Indicators (KPIs) such as reliability, end-to-end delay, radio duty cycle, network formation time, and so forth [39].

The 6TiSCH architecture should provide the following:

- Defining a protocol by which adjacent nodes in the network can arrange the addition or removal of cells from the scheduling matrix;
- Defining a 6top function to create a communication schedule; establishing logical links, including procedures to support distributed dynamic scheduling, as well as mechanisms for maintaining communication schedules;
- Mapping the communication schedule to multi-hop routes created by RPL;
- Resource management, that is, adjusting the resources allocated to adjacent nodes;
- Forcing the differentiation, that is, different treatment of data flows generated by the application layer and signal messages used by 6LoWPAN and RPL to detect adjacent nodes, respond to topology changes, and self-configure IP addresses.

One of the primary goals in designing IEEE 802.15.4e networking is the flexibility in terms of supporting various PHY implementations, including IEEE 802.15.4g. The IEEE 802.15.4g standard represents a revision of the IEEE 802.15.4 from 2015 and defines the PHY specifications for outdoor networking (Wi-SUN networks) [40]. The frequency bands are 868 MHz (Europe), 915 MHz (USA), and 2.4 GHz ISM (global). The compulsory modulation scheme applicable to the PHY is Gaussian Frequency Shift Keying (GFSK), with a transmission rate of 50 kbps, while other modulation types are optional. Combining different parameter values (speed, bandwidth, etc.), this standard offers numerous options for PHY, and achievable speeds ranging from 6.25 kbps to 800 kbps [40]. IEEE 802.15.4g devices can exchange frames up to 2047 bytes. Another amendment of IEEE 802.15.4 is implemented in the IEEE 802.15.4k standard, which defines two additional physical layers [41].

**Figure 1:**
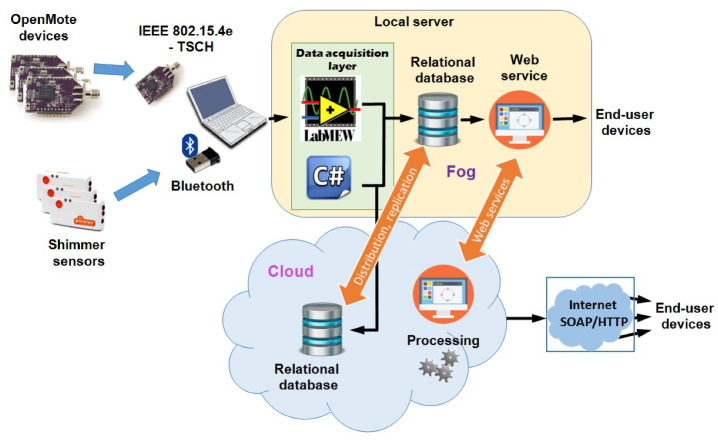
Heterogeneous Internet of Things (IoT)-based architecture for remote healthcare monitoring. Reproduced from [16].

New IoT architectural solutions involve highly dynamic environments with frequent reconfigurations and changes. Therefore, the network design requires careful selection of the topology and architecture, as it critically affects the ultimate transmission performance. Heterogeneous sensor networks can improve overall system performances. In Reference [16], authors propose a heterogeneous IoT architecture for remote health monitoring (RHM) based on Bluetooth and IEEE 802.15.4e-TSCH wireless connectivity. The RHM system encompasses Shimmer physiological sensors with Bluetooth radio and OpenMote environmental sensors with IEEE 802.15.4e radio [42], as illustrated in Figure 1. Network nodes continuously sample and process raw information, sending data to the gateway. The proposed architecture enables data collection from multiple sensors (both on-body and environmental) to process and extract useful information about the current state of the patient, and also about the environment in which a patient resides. Biomedical sensors obtain continuous information about vital signs, whose samples should not be lost. On the other hand, environmental sensors obtain information at a relatively lower frequency compared to biomedical sensors. The data acquisition layer involves gathering signals from sensor nodes and preparing them for storage, analysis, and presentation. This system architecture collects data measurements in a developed relational database to implement a Fog node for quick data analysis as well as in a remote server in the Cloud. Alternatively to using relational databases, it is also possible to store the data in files or use NoSQL databases that can, in the same way as a relational database, reside on the local server (Fog node) and/or in the Cloud.

Machine Type Communications (MTC), Software Defined Networking (SDN) and Network Function Virtualization (NFV) are becoming new frontiers for supporting the emerging IoT applications. SDN introduces a control plane that implements logical functionalities of the network and a data plane to manage the actual transmission of user data [43,44]. The interface between the two planes is provided by an Application Programming Interface (API), called the southbound API, while applications access the control plane via the northbound API. The SDN controller manages the control plane, defines network logic functions based on data plane status (via the southbound API), while the network application requests are routed via the northbound API. With such an organization, SDN enables the adaptation to the available network resources, which provides better flexibility, programmability, and self-configurability [45,46]. NFV implements hardware functions within software applications, resulting in more efficient resource management. In recent years, edge/fog computing technology has been rapidly evolving, as an extension of cloud functionality at the ends of the network [47,48]. Areas of application include geo-distribution, mobility and localization support, and so forth. It also allows the network environment to be adapted to current application requirements or user needs, greatly simplifying network management and resource allocation. The extremely large amount of data generated in IoT networks involves the application of big data technologies as well as advanced data storage and analysis techniques. At the same time, ensuring data security is becoming one of the key elements in the implementation of current IoT networks. As LR-WPAN for smart healthcare usually carries data with sensitive personal or medical information, secure cryptographic protocols should be implemented to protect the data. In Reference [49], one possible solution for a secure bootstrapping scheme applied to WPAN is proposed. A new message structure, named integrity guaranteed message (IGM) structure for IEEE 802.15.4 physical layer is designed. The IGM structure guarantees the message data payload cannot be modified by an adversary without being detected by the receiver. This scheme prevents the man-in-the-middle attack during the key agreement process without utilizing any out-of-band channels.

In addition to the LR-WPAN technologies and standards, in recent years LP-WAN technologies have been developing rapidly, such as LoRaWAN, Sigfox, NarrowBand-IoT, Thread, Random Phase Multiple Access (RPMA), Machine Type Communications (LTE-M). These technologies are increasingly used in IoT applications, particularly due to the ease of implementation, energy efficiency, robustness, support for a large number of devices and long-distance transmission [50].

### 2.2. LP-WAN Standards and Technologies

LP-WAN technology has been developed to address the requirements of low-power long-range applications. More specifically, applications that require a range of a few kilometers and data-rates as low as a few tens of bps. Given this, LP-WAN networks must ensure that ultra low-power operation of the object is performed to extend the battery life of the device. The activity level of these devices may vary depending on the healthcare application and this has to be taken into consideration when designing such a system. One of the design goals is to optimise operation for maximum battery life without compromising reliability. Furthermore, it is crucial that the costs must be kept as low as possible to ensure mass deployment. This means that simple architectures and simple protocols are employed while maintaining the levels of reliability required by a healthcare type of system. Another important aspect to ensure the successful operation of such a technology is the network infrastructure that needs to be deployed at a national and international level to provide a seamless operation to users/patients with chronic health issues. The network might be state-operated or privately operated. Either way, adding the infrastructure and adding a new object should be a simple and low-cost procedure. Another critical aspect of such a network technology is security, especially when applied in the healthcare sector. As data is transferred between users and the network, this can provide the opportunity for spoofing and jamming, putting in danger lives, and corrupting the healthcare system. This subsection presents a number of LP-WAN standards, describing their main features and how these can be employed to enable some healthcare monitoring applications.

#### 2.2.1. LP-WAN Technologies in Licensed Bands

Several LP-WAN technologies have been designed to operate on licensed bands. These technologies may benefit from the dedicated spectrum allocated to them thus offering reliable communications supporting a number of healthcare applications. This can ensure that there will be enough resources for critical healthcare applications to operate.

- LTE CAT-M (for MTC): this LP-WAN technology supports the IoT connectivity through lower device complexity. It is allowed to reuse the LTE installed base stations giving it the ability to provide extended coverage within the existing infrastructure. An extended battery lifetime is supported by CAT-M while the price of the modem is about 20–25% lower than the Enhanced General Packet Radio Service (EGPRS) modems [51]. LTE CAT-M can benefit from the security and privacy the current mobile networks provide. This may be particularly useful for a large scale roll-out of healthcare devices operating on CAT-M. It can operate with 1.4 MHz of bandwidth, unlike the LTE user equipment that requires 20 MHz. CAT-M can support downlink and uplink speeds of up to 1 Mbps. This can be particularly useful for healthcare monitoring applications requiring higher than NB-IoT supported data-rates such as the ones listed in Table 2.

- LTE CAT-N (for NB-IoT): this is the Narrow Band IoT that has been designed to support the operation of WSNs in legacy cellular networks. As some of these types of networks are becoming obsolete, NB-IoT service may prolong their life through a profitable business plan such as offering healthcare-related services. NB-IoT systems may be deployed as stand-alone networks. They may also operate under the LTE guard band. NB-IoT systems employ reduced bandwidth of only 180 kHz thus they may support limited data-rates. They do however provide extended coverage and reduced complexity while keeping the power consumption as low as possible. This could also become the platform for some remote healthcare monitoring applications that do not require high data rates such as body temperature, blood saturation, continuous glucose monitoring (CGM), and so forth.

- EC-GSM-IoT (Extended Coverage - GSM - Internet of Things): this standard is operating in the GSM bands and it is based on eGPRS to deliver high capacity, long-range, low energy and low complexity cellular system for IoT communications. This technology can make use of the legacy GSM infrastructure thus extending its lifetime. This can be achieved with a software upgrade ensuring extended coverage and accelerated time-to-market. It can operate with 200 kHz of bandwidth providing a maximum UL/DL of 240 kbps. Battery life is suggested to last up to 10 years. Like with the NB-IoT technology, EC-GSM-IoT is suitable for low data-rate healthcare applications. It supports strict delay budget making it more suitable for real-time critical applications.

#### 2.2.2. LP-WAN Technologies in Unlicensed Bands

Some LP-WAN technologies have been designed to operate on ISM bands. These technologies, may capitalise on the free spectrum access and use it to provide a number of healthcare applications. This can be particularly useful when dealing with non-critical healthcare applications since the devices may have to share the spectrum with many other devices.

- LoRaWAN is a network protocol characterised by its physical layer technology that modulates signals employing a proprietary spread spectrum technique operating in the sub-GHz ISM band. The LoRaWAN architecture has been designed in such a way to mainly support the operation of battery-powered fixed or mobile devices. Hence, power consumption is of great importance for such networks. LoRaWAN is implementing a star network topology where a number of gateways handle the received packets. It supports three types of devices and how these effectively communicate with the network. For example, Class A devices implement all basic set of features such as bi-directional communication between the device and a gateway. Uplink messages are randomly transmitted while for receiving, the device opens two receive windows at specified times (1s and 2s) after an uplink transmission. Class B operates similarly but on scheduled listening windows and Class C also supports low latency bi-directional communication by keeping the receive window open at any time. LoRaWAN combines Forward Error Correction (FEC) and spread spectrum technique to improve the receiver’s sensitivity. It supports data rates of up to 37.5 kbps [52] and coverage of up to 30 km [53] depending on the transmission parameters such as spreading factor, transmit power, channel bandwidth, and environmental conditions [54].

There are several examples in literature that evaluate the performance of LoRaWAN such as the case in Reference [55], where the application of LoRa on vehicular communications is explored. More specifically, two different scenarios employing LoRa technology were studied. The first scenario presented the Vehicle-to-Infrastructure (V2I) architecture. V2I was deployed to connect a LoRa base station with vehicles equipped with a LoRa end-terminal. Results showed a great performance of the system, with coverage ranges of about 10 km in a suburban scenario. The second scenario involved Vehicle-to-Vehicle (V2V) communication. The results for the second scenario indicated a coverage range of about 6 km. Such studies do open the opportunity for developing novel services and applications in other areas such as healthcare thus contributing to the development of the IoT ecosystem.

- SigFox has partnered with operators to build a network infrastructure consisted of its proprietary base stations. Its operation is based on Differential Binary Phase-Shift Keying (DBPSK) and GFSK while using just 100 Hz of bandwidth requiring a total of 192 kHz of spectrum for its operation [18]. The payload is restricted to 12 bytes at 100 bps limited 140 messages per day. SigFox operates on the 902 MHz band in North America and 868 MHz in Europe. Data generated is tunneled through the SigFox cloud servers and is available to the end-users through a web interface and an API provided. SigFox could be potentially used for healthcare applications although its very limited features will probably restrict its applications to use-cases associated with actuating and monitoring devices that are not life-critical and only a small number of actions are required on a daily basis [56].

- WEIGHTLESS supports a two-way connectivity with two non-proprietary physical layers employing PSK/GMSK and Offset-Quadrature Phase Shift Keying (O-QPSK) modulation using spread spectrum [57]. Every single channel features a very narrow bandwidth of 12.5 kHz [18]. It uses FDMA+TDMA multiple access connectivity for optimal uplink-dominated traffic coming from devices with a moderate payload size. Like most other LP-WAN technologies and standards, WEIGHTLESS is operating on the license-exempt sub-GHz ISM/SRD bands. It supports frequency reuse in large-scale deployments and an adaptive rate ranging from 625 bps to 100 kbps. To maximise network capacity, WEIGHTLESS also support power control in both uplink and downlink. In terms of security, it is implementing AES-128/256 encryption.

- INGENU, unlike other proposed proprietary LP-WAN technologies, operates at 2.4 GHz. Operating in the ISM bands ensures higher market penetration as the spectrum regulations are less strict across the globe. In the uplink, INGENU is using an RPMA Direct Sequence Spread Spectrum (DSSS) as its physical access scheme. This scheme enables multiple transmitters to share the same time slot but in such a way where RPMA reduces overlapping between transmitted signals. Since the 2.4 GHz band has 80 MHz of spectrum, a single RPMA channel occupies up to 1 MHz. It is therefore possible to have 40 channels worldwide with 1 MHz buffer channels to each side. The data-rate supported by INGENU is 78 kbps for uplink and 19.5 kbps for downlink. The range in a rural environment has been set at 15 km and for urban 3 km [58]. It supports both 16B hash and AES 256-bit security encryption.

Apart from the IEEE 802.15.6 standard described in Section 2.1, there is also the IEEE 802.15.4w which is a LP-WAN extension to the IEEE 802.15.4 LECIM PHY layer. It has has been developed by IEEE for low-rate wireless networks. The standard uses LECIM Frequency Shift Keying (FSK) and it operates on both licensed and unlicensed bands. Transmit powers are of the order of 10 mW, while they can achieve long-range communication, typically 10–15 km in rural areas and deep in-building penetration in urban areas. IEEE 802.15.4w also aims to optimise its operation through harsh channel conditions where a significant number of packets are destroyed by interference in densely ISM bands. This can be achieved by introducing Frequency Hopping Spread Spectrum (FHSS) where one packet is split into multiple radio bursts. Further to this, the standard will employ low-rate convolutional and Low-Density Parity Check (LDPC) codes to optimise its data recovery of interfered bursts [59]. The payload bit-rate has been suggested by the IEEE 802.15.4 TG4w to be typically below 30 kbps.

Another IoT standard that can be used for providing a relatively long-range coverage, is IEEE 802.11ah. IEEE 802.11ah has been designed to enable WLANs to provide connectivity to thousands of low-capability M2M devices featuring sporadic traffic characteristics. IEEE 802.11ah operates in the sub-1GHz band while it provides data rates from 0.15 Mbps to 7.8 Mbps depending on the size of its channels. The values of the bandwidth per channel for IEEE 802.11ah are 1, 2, 4, 8 and 16 MHz. The packet length is set 100 bytes. Its typical range could be up to 1000 m. The transmission technique is employing is OFDM while it operates on a single-hop topology. The standard aims at low-power operation to ensure battery operation lasting several months to years. Given its operating characteristics, and based on the communication parameters of medical sensors listed in Table 2, IEEE 802.11ah can potentially become the de-facto platform for BANs and applications such as ECG, SpO2, blood pressure, and so forth.

The technologies and standards presented above can be used alone or combined to provide connectivity to a large number of sensors addressing their unique traffic characteristics. Table 2 lists a number of healthcare applications and their operating requirements obtained from References [10,56,60,61,62,63,64,65]. These parameters are the data transmission rates, energy consumption, and the latency required for ensuring the smooth operation of the sensor node. Based on these values, it is possible to choose the most appropriate communication technology platform.

### 2.3. Security and Privacy for Smart Healthcare

Security considerations for IoT-based smart healthcare communication, whether these are in-body, on-body, or off-body communications, match those of any other type of communication device handling sensitive data. Security challenges such as reliability and data privacy must be addressed in order to improve confidence. Common threats, steaming from passive or active attacks, can result in confidentiality attacks, access control attacks, integrity attacks, authentication attacks, and availability attacks [66]. Furthermore, eavesdropping, evil twin access point, and man-in-the-middle are typical types of attacks that can challenge the confidentiality of the system while Denial-of-Service (DoS), Replay attack or Frame Injection attack challenge system’s integrity. Radio frequency (RF) jamming, beacon flood, and Association/Authentication Flood can severely limit the availability and undermine the safety and privacy of the patient. In some cases, malicious attackers may employ other types of extreme and sometimes novel ways to acquire sensitive data. According to Reference [67] eavesdropping on handwriting can be achieved using nearby mobile devices based on audio signal processing and machine learning. Authors claim that there is a good chance to achieve 50–60% of word recognition under certain conditions. This work has been extended to also include a hand motion tracking method to enhance the performance of the eavesdropping, thus improving performance to 70–80% [68]. All these types of attacks show that security and privacy for smart healthcare are of extreme importance for the viability of smart healthcare.

Privacy in terms of smart healthcare may be viewed as the right of the individual patient to be able to make decisions about how personal information is shared. It also means that any information shared within the healthcare network along with other personal information must be kept private. Some laws that ensure that the patient has every right for privacy but in practice, this is challenging given the scale and complexity of the network that supports the operation of smart healthcare applications. Therefore, privacy may be at risk when security is breached. Data security and privacy issues have become the biggest concern for people using the smart health (s-Health) [69] ecosystem, where patients expect that their s-Health records (SHRs) [70] may be only accessed by authorised professional healthcare staff. According to Reference [70], in the case where traditional access control techniques are adopted, either data security is violated or only coarse-grained access policies are allowed. To mitigate this issue, authors introduced a Privacy-Aware S-Health access control system (PASH). PASH is based on Ciphertext-Policy Attribute-Based Encryption (CP-ABE) scheme and attribute values of access policies are hidden in encrypted s-health records (SHRs) and only attribute names are revealed. The authors added an efficient decryption test that needs a small number of bilinear pairings, before full decryption to improve efficiency. Theoretical analysis and experimental results indicated that PASH is more secure, efficient, and expressive than existing schemes.

The need to address any security and privacy considerations associated with the operation of healthcare devices has become a necessity. Recent advances in healthcare technologies increasingly offer many functions and features for medical sensors, based on software implementation (e.g., software control of pacemakers). Using software to operate such devices made it possible to offer more complex functions, as well as features that increase, improve, revise the usability of the device. Such functionality involves remote access and control of the device, cloud based interfaces, remote enabled data collection, as well as Over-the-Air (OTA) update of the software. Providing wireless connectivity to such sensors may pose numerous challenges to developers with regards to their security and safety. Security attacks to any wearable, implantable medical device can result in loss of privacy of the patient (e.g., loss of their data) or loss of control of the device. This can effectively lead to the unintended operation of medical devices [71]. It is therefore imperative to strengthen the protection of sensitive data and patient privacy data. Table 3 lists some of the most common threats/attacks that smart healthcare systems might be exposed to. It also provides a brief description on the techniques that can be used to mitigate these threats/attacks.

The need for improved cybersecurity for medical devices has been recognised as a priority by the FDA. FDA issued its first safety-related communication regarding the risks associated with cybersecurity back in 2015. Furthermore, the FDA warned clinicians that the Hospira external drug infusion pump was compromised with security flaws [74]. Similarly, in 2016 Johnson & Johnson announced that their OneTouch implantable insulin pump system was exposed to hackers through a security vulnerability where it allowed them to overdose diabetic patients with insulin. Another major vulnerability that ended in an unprecedented FDA recall of 465,000 devices was the St. Jude Merlin@Home pace-maker monitoring system. The flaw here was that the St. Jude Merlin@home transmitter, specifically the “inductive” and RF models, were vulnerable to “Man-in-the-Middle” (MITM) attacks. This could potentially allow attackers to compromise the device remotely as well as eavesdrop on the communication [75].

The authors of Reference [76] present a number of security threats against Implantable Medical Devices (IMD). This is particularly interesting as these threats can be generally projected to WBAN type of medical devices. According to Reference [76], among many types of security properties, confidentiality is at risk when eavesdroppers monitored the radio channel (401–406 MHz) when the IMD and the programmer communicate. Furthermore, availability could be compromised when an IMD is rendered inaccessible through the blockage of the radio channel (active jamming). Cryptographic mechanisms can be employed to protect the wireless communication channel and the records stored in the device. Due to the resource limitations of IMDs discussed in Reference [76], the current generation of implants, are based on symmetric-key approaches that suffer from the key distribution problem. In a massive scale deployment of such wearable or implantable medical devices, using an IoT based communication technology, attacks are expected to increase as different communication standards encompass different vulnerabilities. In the foreseeable future, the network connectivity of IoT-based healthcare wearable/implantable solutions might be provided by the 5G networks.

5G mMTC (Massive Machine-Type Communications) may be required to connect millions of such healthcare type of sensors to the network. Such sensors will be exposed to a number of threats and people’s lives might be at risk. 5G networks are and will be in danger for cyber espionage as it is estimated that 5G networks will experience many more attack vectors compared to previous 4G networks. With the technological advancements of Quantum Computing (QC), it is expected that these threats will increase given that the current public-key cryptography is vulnerable to its powerful processing capabilities. It is therefore imperative that action must be taken at a very early stage to secure vital communications against snooping. Quantum Key Distribution (QKD) is a technology that can enhance the level of security of future 5G (and beyond) networks that will be used to secure the distribution of symmetric encryption keys. This will be achieved by allowing the exchange of a cryptographic key between two remote entities with absolute security [77]. The European Quantum Flagship by the EC has already planned a road-map towards the commercialisation of all quantum technologies including the QKD. The plan is that within 6-10 years, advanced QKD and Quantum Random Number Generator (QRNG) systems will be available for IoT and 5G networks (among other services) in a trusted-node network. This type of network will be based on fiber, free space, and satellite links. The end product will effectively offer end-to-end security over trusted nodes. The network will expand across Europe using (optical) repeaters and satellites between countries. A similar approach was undertaken by individual countries such as China, the United States, Australia, South Africa, Japan, and so forth, where a number of trials have been conducted [78].

Providing advanced cybersecurity technologies and architectures is imperative for delivering safe, secure, and trusted smart healthcare. Without them, smart healthcare IoT-based systems will not be able to reach the market.

## 3. Emerging Trends and Applications in Smart Healthcare

### 3.1. Application Domains and System Architectures

There is no doubt that IoT technologies are transforming the area of healthcare by enabling new ways of interaction between doctors, patients, and medical devices. In particular, the application of IoT in healthcare can bring many benefits to all involved parties. Depending on the target end-user, the IoT healthcare applications can be grouped as follows:

- **Healthcare IoT for Patients** provides patients with personalized healthcare utilizing different wearable devices such as activity trackers [79], inertial sensors [80] heart-rate and blood pressure monitoring sensors [81], glucose-level meters [82]. These devices are used to track patient’s healthcare parameters in real-time and even perform local data analysis in addition to storing the historical data in the cloud. Based on these measurements, the patients are given personalized suggestions on how to improve their medical condition, while at the same time potentially life-threatening conditions can be detected on time. This is expected to have a strong impact on people’s lives, especially for those living alone and improve their sense of health security.
- **Healthcare IoT for Doctors** provides them with timely information about the health conditions of the patient in the hospital. Besides, it also enables them to keep track of the health condition of the patients in the home environment, which was not possible until the advent of healthcare IoT. In addition to the aforementioned benefits, the large amount of collected data regarding the patient’s health condition enables the use of advanced machine learning techniques. These can be used to identify the effectiveness of the treatment, learn more about the illness itself, and monitor its progress.
- **Healthcare IoT for Hospitals** is expected to propel the further use of IoT in the healthcare field. In particular, by tagging different hospital assets such as defibrillators, respirators, and monitoring equipment, their location can be tracked in real-time. Besides, if the medical staff location is also being tracked, there also exists a possibility to optimize their engagement and easily locate them in the case of an emergency. Finally, the analysis of such data could provide the hospitals with new insights on how to improve the overall organization and use of resources.

**Figure 2:**
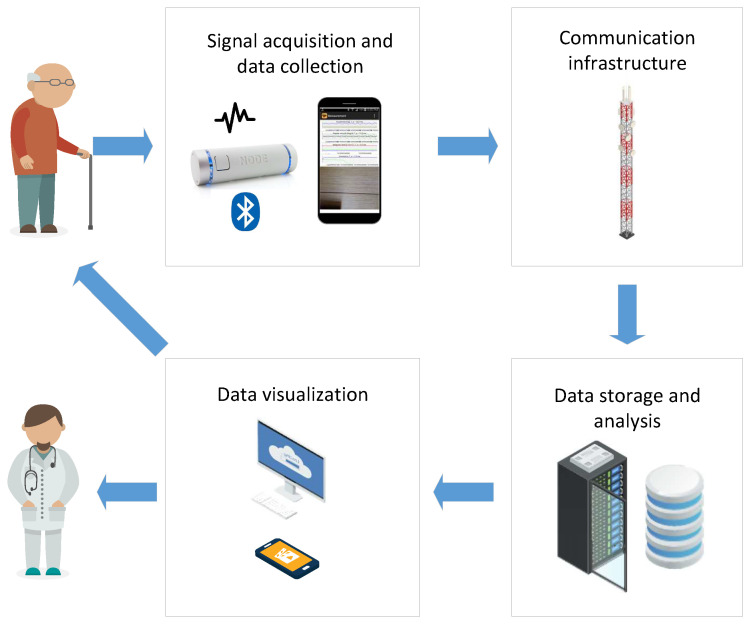
Architecture of IoT healthcare system.

In order to support the aforementioned applications, the IoT healthcare system shall be designed to be flexible enough. In Figure 2, we show the general architecture of the IoT healthcare system. It consists of the following blocks:

- **Signal acquisition and data collection:** the goal is to sense and transmit different measurements of patient vital signs, as well as environmental data that may be relevant for the analysis. It usually consists of one or more biomedical sensors such as heart-rate sensors, blood pressure, temperature, and so forth, which send the measurements via a wireless connection to the subsequent data collection unit. The signal acquisition components shall be designed to be portable, non-intrusive, and energy-efficient in order not to interfere much with the patient’s activities and at the same time provide high-quality data about the patient’s health condition. Besides, this block can also perform local pre-processing before sending the data to the main data storage and analysis block. Depending on the availability of internet connection at the given location, this module is usually equipped with mobile, Wi-Fi, or Ethernet connectivity unit.
- **Communication infrastructure:** aims to transfer the data from the signal acquisition and data collection block to the subsequent data storage and analysis. Depending on the availability of communication networks and the requirements of the underlying data (bit-rate, latency), this block could use cable Internet, Wi-Fi, 4G, and so forth.
- **Data storage and analysis:** is usually implemented as a cloud server, due to cost-effectiveness, ease of configuration and scalability. Its main purpose is to collect and store data from different data collection units deployed in the patient’s home, perform data analysis by using different signal processing and ML algorithms, and enable access to such data by different users. In addition, strong security mechanisms (data encryption, secure communication, access control) shall be enforced. This is to ensure data privacy protection and prevent any possible misuse of highly sensitive healthcare data.
- **Data visualization:** represents the final block which is in charge of communication of the results of the analysis towards the end-user. This block is usually implemented as a web, desktop or mobile application, which may be aimed at either healthcare practitioners or the patient itself. In such a way, the patient is informed about its healthcare condition in a timely manner. This component shall be implemented having in mind best practices in user interface design and usability, to ease adoption by the end-users.

### 3.2. Crowdsourcing/Crowdsensing for Healthcare Applications

With the emerging achievements of the digital era, big data becomes an unavoidable paradigm of everyday life. The formal big data definition can be a subject of discussion [83], but its basic parameters such as huge volume, high (near real-time) velocity, and diverse variety are foundations that align with the needs of contemporary science. The potential utility of acquisition, storage, and analysis of massive data quantities, that is, big data, has been recognized in healthcare as well. Recent surveys justify the growing big data implementation in medical systems. It was reported that the number of scientific papers on big data in healthcare follows a parabolic increase in the MEDLINE database—while no papers were reported in 2010, almost a thousand ones were found only eight years later [84]. In the PubMed database, within the same time slot, the number of papers increased from 25 to 350, as reported in Reference [85]. The abundance of research projects supported by the EU, systematized in Reference [84], confirms that big data presents many significant opportunities for healthcare innovation.

However, data gathering is still an uncharted field. One of the possibilities related to wireless networks is crowdsourcing/crowdsensing.

The term crowdsourcing appeared in 2006 [86], loosely meaning “data, tasks, or projects collected from a large number of paid or unpaid people”. The applications in health followed immediately. The first survey on crowdsourcing in healthcare appeared in 2013 [87]. The author started with 231 peer-reviewed articles, but, following the strict selection criteria, analyzed 21 papers, with a conclusion that crowdsourcing in health can be beneficial for problem-solving, data processing, surveillance/monitoring, and surveying, the letter two issues related to the data acquisition.

The tenth crowdsourcing anniversary was marked by a general survey [88] that included numerous medical applications. The importance of crowdsourcing in health is emphasized in a review that followed immediately, focusing the medical applications [89]. The authors found 996 papers but analyzed 286 ones that fulfilled the stated criteria. The most exhibited 66 papers are outlined and briefly described in the table. Another crowdsourcing health-related systematic review [90] included 202 studies with conclusions similar to Reference [87]. It is interesting to note that the growing number of both crowdsourcing and big data healthcare articles follows a similar and synchronized trend.

Mobile crowdsensing (MCS) can be regarded as a subset of crowdsourcing where data harvesting is performed by sensors, without (much) human involvement [91]. It exploits the fact that the majority of the human population owns smart and wearable mobile devices equipped with sensors, ready to record, process, and transmit a great variety of data. Wearable devices induce already mentioned constraints of reduced power resources. In a crowdsensing environment, it can be resolved either with the analytical tools designed for reduced consumption, for example, Reference [92], or designing the platforms for supporting emerging computation-intensive applications on capacity and resource-constrained mobile devices, such as Reference [93].

The term “mobile crowdsensing” was proposed in 2011 [94]. Contrary to crowdsourcing, the initial crowdsensing typology included environmental, infrastructural, and social applications [95], with the health issues brought into the focus only recently. Despite the late arrival, a survey of the healthcare crowdsensing articles has already appeared [96], with 1705 articles found according to the title and abstract, but with 13 papers included in the study due to the rigid exclusion criteria.

The heterogeneity in platforms, frameworks, and architectures of mobile crowdsensing was considered in Reference [97]. The authors proposed a Reference Architecture strictly within the bounds of mobile crowdsensing with particular attention dedicated to the healthcare domain. The illustrative example was devoted to patients suffering the Tinnitus, a disorder of unknown origin that causes a ringing sound in ears. Another approach applies machine-learning techniques and features extracted from the electrocardiogram (ECG) to inform the hypertensive patients about their status during various activities [95].

MCS is a voluntary activity and subjects need motivation, otherwise, they cease to be involved. Healthcare MCS systems have an advantage, as subjects with specific health conditions are interested in their well-being and more willing to participate. Nevertheless, several incentivization schemes for crowdsensing in healthcare have been proposed. In Reference [98], the incentive mechanism for collecting health-related data was presented, based on the best response dynamics in which participants have incomplete information on other participant’s costs. A game-theoretical multi-window framework is introduced where a stable solution is achieved after several iterations. In Reference [99], the incentive mechanism is based on a reverse combinatorial auction. As a metric, it implements the Quality of Information (QoI) [99]. The QoI definition depends on the application. If the acquired data is an image, the QoI is related to its quality (resolution, contrast, sharpness). In the case of 1D signals, the factors that degrade QoI can be poor sensor quality, noise, and lack of sensor calibration.

## 4. Technical Challenges in Smart Healthcare

### 4.1. Designing IoT for Smart Healthcare

Since connected medical devices collect patient’s vital parameters, it is of utmost importance to ensure their performance and reliability. In particular, any performance degradation and reliability loss may result in an elevated risk for patient’s safety and lead to potentially life-threatening conditions, depending on the particular IoT application. The design and implementation of these devices pose many technical challenges that have to be solved before they can be put on the market.

One of the challenges that has received particular attention is data security and privacy in healthcare IoT. Namely, devices used in healthcare collect and transmit highly sensitive personal data. These data, if accessed without permission, can be misused by hackers to compromise the patient’s medical record, issue a fraudulent claim in the patient’s name to the insurance company or cause dangerous conditions (e.g., in the case of an insulin pump). This challenge is further complicated since most of the IoT devices lack standard data protection mechanisms, due to a highly competitive market where the vendors are forced to significantly shorten the time necessary for development from the prototype to the final product.

**Figure 3:**
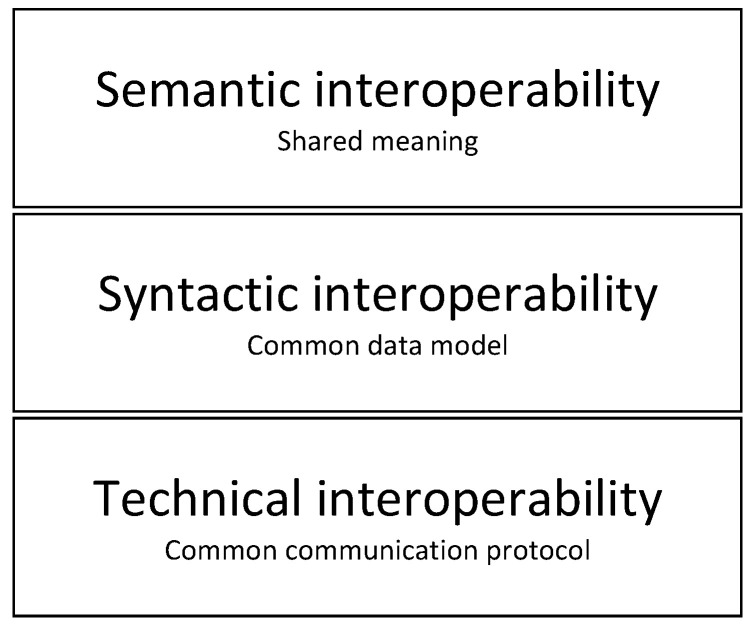
Layers of interoperability.

Another challenge that is present in the whole IoT ecosystem, and not only in the healthcare sector is interoperability. It can be defined as the ability of an ICT system to operate with parts of other systems. The main reason why this is still a challenge is that device vendors have not achieved a consensus in relation to common communication protocols and standards. Figure 3 presents the Interoperability model. As can be seen, the model is composed of multiple layers that represent the aspects to be fulfilled in order to achieve full interoperability among different systems:

- Technical interoperability layer ensures that systems use common communications channels and protocols (e.g., ZigBee, NB-IoT, LoRaWAN, etc.). This layer represents the lowest level of interoperability which is required to facilitate minimal interoperability conditions. Usually, TCP/IP is used as a common solution for technical interoperability. However, in constrained applications where energy consumption has to be reduced, other more energy-efficient protocols are used.
- Syntactic interoperability layer further builds on the previous layer by ensuring that a common data format is used for the exchange of messages between different systems. At this interoperability layer, two or more systems can interpret the content of exchanged messages.
- Semantic interoperability layer ensures that the meaning of the data is represented semantically, categorized and linked to other semantic data. This layer ensures that systems can combine received information with other information sources.

It must be noted that the technical interoperability has been mostly achieved by using well-proven communication protocols. However, higher levels (syntactic and semantic) still remain as open points which will require closer collaboration among the vendors. Although the interoperability model presented in Figure 3 does not explicitly deal with the security and privacy, these are implicitly considered at all the aforementioned layers. In particular, the security schemes are implemented from the technical interoperability layer (communication protocols) up to the syntactic and semantic interoperability layer where proper authentication and authorization schemes have to be implemented.

IoT healthcare systems can potentially consist of a large number of devices deployed in a patient’s close environment and on-body sensors. Consequently, the storage and analysis of the massive amount of data generated by such devices pose a specific challenge. Although the advent of cloud computing and the availability of on-demand computing resources have alleviated this issue, there is still room for improvement in this area. In particular, the need for fast storage and query of time-stamped data can be fulfilled by using more advanced database engines. As an example, InfluxDB [100] is a time-series database designed to support high write and query loads, as well as aggregation and expiring old data. Once the challenge of data storage and retrieval is solved, there still exists a need to perform automatic analysis on patient’s data and provide meaningful results and recommendations. Data analysis is usually performed by using machine-learning techniques that take advantage of such a large amount of available patient data to provide more accurate results and even make conclusions not possible by using other more simple data analysis techniques. In particular, ML can be used to aid healthcare practitioners in the diagnosis of diseases which may be hard to diagnose. This includes cancer at an early stage by analyzing MRI and microscopic images. Moreover, it also provides the efficient personalized medical treatments by coupling the status of patient health with predictive analysis.

### 4.2. Crowdsensing/Crowdsourcing Challenges

The crowdsourcing and crowdsensing are praised in unison for being beneficiary for patients, researchers, and health-care providers, in all the articles, reviews, and surveys [101]. Critics, if any, are mild and vague. The only sincere doubt, but considering the statistical soundness of the collected data, is raised by a medical doctor [102] who points out the limitation of self-reported data, sample selection bias, data homogeneity, and the fact that institutional review boards generally disapprove such research.

Statistical soundness, examined in Reference [102], is not a major problem in medical crowdsensing data harvesting. The final beneficiary is a patient, so her/his safety should be the greatest concern. The safety can be perceived from two different viewpoints. The first one includes the privacy, confidentiality, and integrity of the patient’s data. It is a well-covered field, as presented in Section 2.3, with contributions spreading mobile crowdsensing in healthcare as well [103]. The second safety viewpoint is the reliability of acquired data: privacy is of no use if the recorded signal is erroneous and gives distorted information of the patient’s well-being and wrong input to the database. This important issue is seldom addressed. It is a paradox that data transmission is subject to the firm regulations of communications regulatory bodies, protected by the latest cryptographic tools, while this very same data is acquired using the procedures that still reside in a twilight zone.

The problem with signal acquisition is twofold. The first issue is the lack of standards, a characteristic of cardiovascular data acquisition. The cardiovascular data are dominant in smart wearable devices, the core of crowdsensing data gathering, so this problem deserves special attention.

In contemporary equipment, ECG sampling frequency can get any value between 80 Hz and 1000 Hz. These frequencies satisfy the constraint of the Sampling theorem [104], but the obtained samples are just the first phase in signal reconstruction. The second phase is ideal low-pass filtering, given by an interpolation equation $ECG\left(k\Delta t\right)=\sum_{n=-\infty }^{\infty }ECG\left(nT\right)\cdot \mathrm{sinc}\left(\frac{k\Delta t-nT}{T}\right)$, where *k*Δ*t*, Δt≪T, substitutes a continual variable “*t*”, *T* is sampling period, *ECG*(*nT*) is a signal sample, and sinc(x) = sin(x)/x [104].

**Figure 4:**
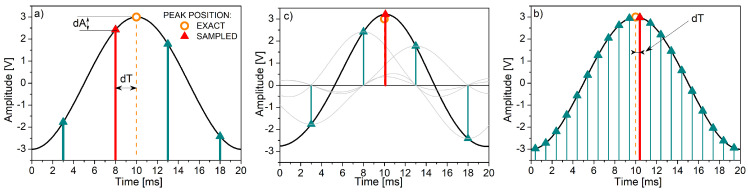
Peak position estimation: (**a**) Source signal (black line) sampled with 200 Hz (vertical lines); the error shift dT between the signal maximum (dashed line) and sample maximum (red line) is equal to 2 ms; dA is a corresponding amplitude change; (**b**) The interpolated samples (black line); gray lines show sin(x)/x interpolating functions; the error shift is reduced, but it still exists (dT = 0.1 ms) as the number of interpolating points is not infinite; (**c**) Source signal sampled with 1000 Hz; the error shift dT is equal to 0.4 ms.

Cardiovascular acquisition equipment, as a rule, provides the results of the first phase - signal samples. This is illustrated in Figure 4. It presents a signal drawn with the resolution of Δ*t* = 0.1 ms, with the goal to find its peak position. If the sampling frequency is equal to 200 Hz (Figure 4a), the samples without the interpolation yield an error shift dT—the time offset between the correct and the sample peak positions. In this example, dT is equal to 2 ms. The finite interpolation of 12 neighboring samples could have reduced the error shift to dT = 0.1 ms (Figure 4b), but it is rarely implemented. The sampling frequency increased to 1000 Hz considerably exceeds the Sampling theorem requirements but provides the error shift equal to dT = 0.4 ms without the interpolation (Figure 4c).

The correct peak detection is necessary because some of the most important cardiovascular diagnostic and prognostic markers are obtained from the R-R interval and heart rate (HR) time series [105]. R-R intervals are intervals between two adjacent "R" peaks in an ECG signal. Heart rate signal is derived from the R-R time series as 60/(R-R), where R-R is expressed in seconds and HR in beats per minute (bpm).

**Figure 5:**
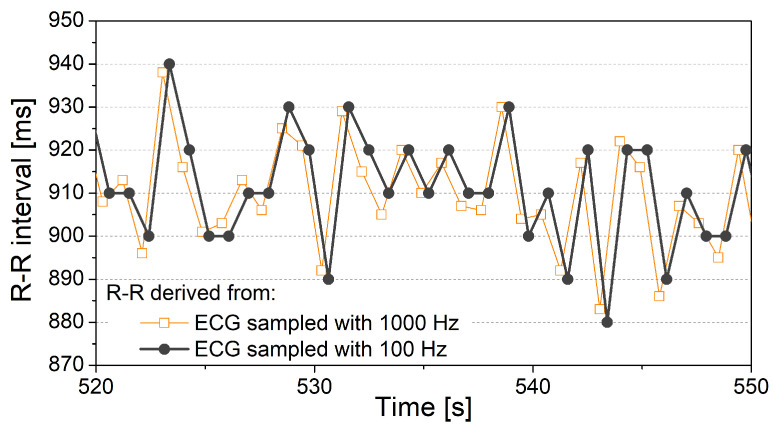
R-R interval time series derived from the electrocardiogram (ECG) signal. If ECG sampling frequency is equal to 100 Hz, the number of different R-R values is only seven (marked by horizontal lines). The ECG signal was acquired by TaskForce monitor (https://www.cnsystems.com/products/task-force-monitor) at Bežanijska Kosa Hospital. ECG sampling frequency was 1000 Hz, then it was downsampled to 100 Hz to show the decrease of resolution in R-R signals. The signal was recorded from a healthy volunteer who signed an informed consent about the participation in the experiment. The protocol was approved by the Ethics review board of the Medical Faculty, University of Belgrade.

Figure 5 shows an example of R-R intervals estimated from the high- and low-frequency sampled ECG signal. The R-R series is first derived from the 1000 Hz sampled ECG signal. Then the ECG signal is downsampled to 100 Hz, and R-R intervals are derived again (Figure 5). Both R-R signals appear to be similar, but the variability of the second R-R time series is considerably reduced—It comprises only seven different amplitude values. Data with just seven different values carry a reduced amount of information. It is particularly undesired if a differential coding is applied, as more than 6% of adjacent samples would have the same value [92]. Equality of adjacent samples would induce a bias in results and prevent the analysis of increasing and decreasing sequences of R-R samples, related to sympathovagal balance and clinical conditions such as hypertension.

The sampling frequency of ECG signals should be large to ensure reliable results if the interpolation is unavailable; and it should be standardized to ensure comparable results. Unfortunately, the most quoted guidelines on cardiovascular data were published a quarter of a century ago [106].

Errors – in biomedicine usually called artifacts – present the second issue. Error elimination is a complex procedure as classical filters cannot be used – they disturb the temporal position of signal samples that can gravely deteriorate the medical parameters. It is especially exhibited in baroreflex studies. Medical society prefers to remove the artifacts manually, a task that is both time-consuming and tedious. A less preferred approach is semi-automatic. For example, most of the artifacts in the heart-rate signal are identified using a filter specifically designed for this purpose [107]. Residual errors are found by visual inspection. This filter, however, is not suited for other cardiovascular waveforms. Stationarity, required for almost all analytical tools, is ensured using a filter also designed for medical signals [108].

**Figure 6:**
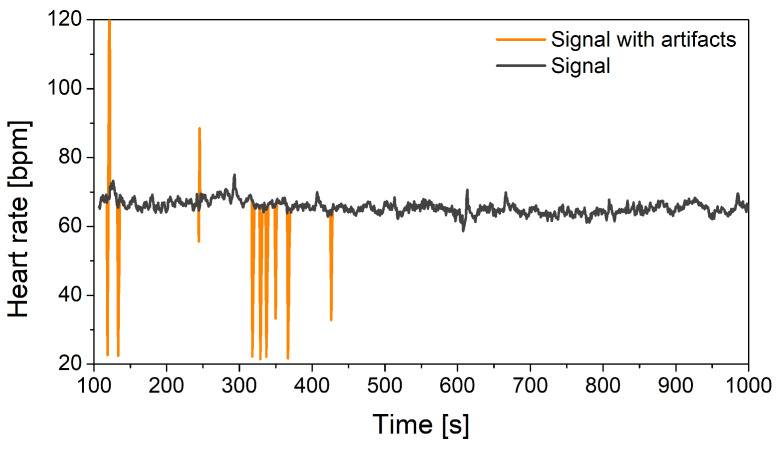
Heart-rate signal expressed in beats per minute (bpm) and typical artifacts. The heart rate was derived from the ECG signal recorded by TaskForce monitor at Bežanijska Kosa Hospital. The signal was recorded from a healthy volunteer who signed an informed consent about the participation in the experiment. The protocol was approved by the Ethics review board of the Medical Faculty, University of Belgrade. The artifacts were identified manually and replaced with the mean value of the nearest correct samples.

Figure 6 shows artifacts in the heart rate signal. Such artifacts are typical for software tools applied for ECG delineation. If uncorrected, the patients’ results can be falsely converted from normal to pathological (and vice versa). For example, a sympathovagal balance (a ratio of low- and high-frequency components) of heart-rate presented in Figure 6 has changed twofold, despite only 0.9% of samples affected by artifacts.

**Figure 7:**
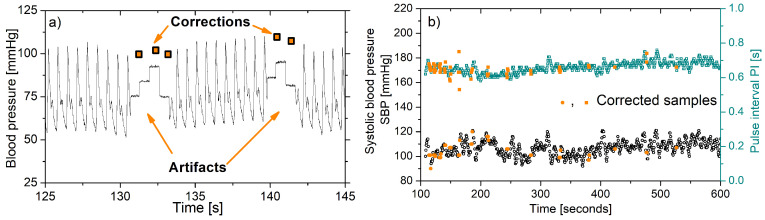
Artifacts in portable blood pressure monitor: (**a**) Typical artifacts with the corresponding automatic corrections (**b**) Systolic blood pressure and pulse interval signal samples with embedded corrections. The blood pressure waveforms were recorded by Portapres monitor (http://www.finapres.com/Products/Portapres) at Bežanijska Kosa Hospital, with 100 Hz sampling frequency. The systolic blood pressure peaks were corrected by the Portapres software. The signal was recorded from a healthy volunteer who signed an informed consent about the participation in the experiment. The protocol was approved by the Ethics review board of the Medical Faculty, University of Belgrade.

An example of a typical software artifact occurring in a portable blood pressure monitor is shown in Figure 7. Although the monitor provides embedded error correction (Figure 7a), the correction is focused to preserve the mean values of extracted parameters (systolic and diastolic blood pressure, pulse interval) and not to recover the true peak positions. Besides, the errors are frequent due to the hardware constraints, with too many corrections (Figure 7b), so the results of a deep analysis are not reliable.

Another source of artifacts in biomedical signals is a non-ideal sensor attachment. A study [109] revealed that out of 523 patients recorded in *ideal* hospital conditions, 115 patients had too many artifacts so their signals had to be discarded. Knowing that in the crowdsensing environment the sensors are not attached by medical professionals and that the patient is freely moving, inducing the friction at the skin-sensor interface, the results can be expected to be worse.

Data reliability could be assessed in a more sophisticated way. In Reference [110], the true information (the truths) among conflicting sources of data is extracted using an optimization framework where truths and source reliability are defined as two sets of unknown variables. The objective is to minimize the overall weighted deviation between the truths and the multi-source observations where each source is weighted by its reliability. This approach is general, illustrated with the weather and stock data, without taking into account the specific properties of biomedical signals. Another big data crowdsensing platform [111], Trust-based Minimum Cost Quality Aware data collection scheme, performs data collection optimization in a malicious environment. Data are collected from data reporters (signal sources) and the trust value of each data reporter is evaluated by the submitted data with a time decay function based on the idea of machine learning. Substituting *malicious* with *erroneous*, and *data reporter* with a *patient*, the method could be easily adapted to identify the unreliable signals. However, this approach is also designed for general applications.

These few examples illustrate the problems the data collection faces—There is still a gap between two different professions. Medical doctors often trust the printout digest of the recorded data, without thoughts that the signal must be visually inspected prior to the decision about the trustworthiness. Even the lack of standards remains unnoticed, as medical doctors work according to the firm medical procedures and do not expect that the properties of acquired data depend on vendors.

On the other hand, the particularities of medical data are beyond the knowledge of the engineers. The engineers are not fully aware of what the data would be used for, and how to modify the classical processing tools to be adjusted to the needs of medical procedures. As an outcome, the applied signal processing tool might not work correctly without the adjustment and the point is that no one would notice. An illustrative example is a simple moving average filter recommended for signal smoothing before the baroreflex estimation; as an outcome, the baroreflex is not estimated from the obligatory adjacent heart-rate samples, but from the samples spread 9 hear-beats apart.

The massive crowdsensing is envisaged for creating huge databases that contain referent medical data, available to researchers and medical doctors. But the true challenge is to establish a firmer interface between the medical and technical experts, to provide the missing standards on sensory data acquisition and to create the guidelines for signal processing procedures. Only then the harvested medical data would deserve the label “big”.

## 5. Conclusions

Although considered as a mature technology, WSN-based IoT healthcare systems still need to evolve in order to fully integrate the following functionalities:

- Interconnection: The ability to enable the efficient and reliable interconnection of network devices, sensors, and people within a single communication infrastructure. Interconnection provides the collection of data from all parts of the healthcare system, thus offering the required functionality and identification of possible system improvements and innovations.
- Information transparency: Transparency should provide operators with access to relevant information needed to make timely and reliable decisions.
- Technical assistance: The system should have the ability to aggregate and visualize information in order to solve problems in a short time.
- Intelligent decision-making: A real-time and interactive decision-making IoMT system should provide accurate diagnosis and treatment services, data fusion from multiple sources, and multimodal ML-based decision-making engines.
- Safety and Security: The ability to deliver highly secure networks that will ensure the safety and privacy of the patients. This will require to employ new design paradigms based on well-founded security practices to build trustworthy systems. Designers must consider security at the very early stages of the design of smart healthcare systems.

Current trends in the development of advanced IoT smart healthcare frameworks are focused on:

- Development of advanced API models, such as REpresentational State Transfer (REST) scalable architecture.
- Innovative cloud platforms that provide efficient extraction of data context and decision making.
- Artificial intelligence, big data, and cognitive systems in the processing of large amounts of data.

Our individual existence and well-being depend on being healthy and when we get sick, receiving individualised, optimal therapy [112]. Furthermore, we need to take control of the increasing costs associated with healthcare (currently 4 billion euros per day in Europe alone) and make sure that our healthcare system is sustainable in the future. There is also an urgency to move from the traditional healthcare approaches, where the majority of our efforts are focused on treating rather than preventing diseases [112]. It is important to be able to diagnose diseases early enough to be able to provide life-saving therapies. A revolution is therefore required that will take advantage of the recent technical developments. Further to this, we need to reconsider how our healthcare system will work in the future and revisit its legal basis, security and privacy regulations, and reimbursement mechanisms. Smart healthcare technologies are gradually becoming an integral part of our healthcare system aiming to provide personalised medicine and provide prevention. Emerging WSN and IoT technologies will form the foundation for smart healthcare, where AI and advanced algorithms will provide reliable support for medical monitoring and diagnosis. The future IoT smart healthcare systems will encompass more powerful and new types of sensors (for example, nanoscale sensors) and advanced communication technologies in terms of coverage, reliability, resource management, security and privacy, as well as data analytics and management. Nevertheless, there are still many challenges and open issues in implementing the advanced and robust IoT healthcare architecture, which future communication standards need to resolve, such as IT security and privacy issues; reliability and stability necessary for critical M2M communication; the overall system integrity and maintenance; prevention of critical conditions and process interruptions, and so forth.

## Figures and Tables

**Table 1:** Low-power wireless technologies for smart healthcare.

Technology	RFID	Bluetooth/BLE	ZigBee	TSCH	Wi-Fi HaLow
Standard	ISO/IEC 15, 693	IEEE 802.15.1	IEEE 802.15.4	IEEE 802.15.4e	IEEE 802.11ah
Frequency band	860–960 MHz, 2.4 GHz	2.4/5 GHz	868/915 MHz, 2.4 GHz	2.4 GHz	Sub-1 GHz
Data Rate	106–640 kbps	1–24 Mbps/BLE: 2 Mbps	20–250 kbps	Up to 250 kbps	150 kbps to 78 Mbps
Energy efficiency	High	Medium; BLE: Very high	High	Very high	High
Transmission range	Up to 50 m	10–100 m/Bluetooth 5.0: up to 250 m	10–150 m	10–150 m	Up to 1 km
Reliability	Medium	Medium/High	Medium	Very high	High
Mesh networking	Yes	No/Bluetooth Mesh: yes	Yes	Yes	No
Typical applications	Patient and medical equipment localization	Wearable healthcare monitoring, data acquisition	Home health monitoring, data aggregation	Healthcare in residential environment, data aggregation	Remote patient monitoring, backhaul aggregation, video streaming

**Table 2:** Communication parameters for medical sensors.

Sensor Node	Data Rate	Sampling	Nodes	ADC	Power Consumption	Privacy Required	Latency
Glucose sensor	<1 kbps	<50 Hz	-	16-bit	Extremely Low	High	<150 ms
[10,56]
Pacemaker	<1 kbps	<500 Hz	-	12-bit	Low	High	<150 ms
[10,56]
Endoscope capsule	1 Mbps	-	2	-	High	Medium	<150 ms
[10,56,60]
Cochlear implant	<1 Mbps	5, 12, 49 MHz	-	-	Low	-	<150 ms
[56]
ECG (12-channel)	72 kbps	<500 Hz	<6	12-bit	High	High	<250 ms
[10,56,60,65]
SpO2	32 kbps	-	-	-	Low	High	<250 ms
[10,65]
Respiration	<10 kbps	-	<12	-	High	Medium	<250 ms
[60]
Blood pressure	<10 kbps	<100 Hz	<12	12-bit	High	High	<150 ms
[10,56,60,65]
EMG (12-channel)	1.536 Mbps	8 kHz	<6	16-bit	Low	-	<250 ms
[60]
Temperature	<10 kbps	-	<12	-	Low	-	<250 ms
[56,60,62]
Blood flow rate	480 kbps	<40 Hz	-	12-bit	Low	-	<150 ms
[56]							

**Table 3:** Security and Privacy–Threats and Actions for Smart Healthcare Systems.

Type of Threats, Attacks	Requirements	Description	Possible Actions
Eavesdropping, Evil-twin access point, Man in the Middle	Confidentiality	Intended users (patients, medical staff or even devices) may only access confidential data. Confidentiality aims to secure this access. Smart Healthcare devices must be able to safely transfer their sensitive data.	Privacy is at risk when confidentiality is bridged. Early detection of such threats is crucial. To mitigate these threats, it is necessary to employ cryptographic techniques for preventing eavesdroppers from intercepting data transmissions between legitimate users.
Insider attack, Replay attack, Frame injection attack	Integrity	Any type of attack that can alter medical data can be catastrophic for a Smart Healthcare system such as a Hospital Information System. Integrity aims to guarantee the accuracy of the transmitted information without any falsification [66].	Detect such attacks as early as possible. All data values must satisfy semantic standards while unauthorized tampering is eliminated [72]. Employ techniques such as digest, digital signatures or watermarking in the case of multi-media data [73].
DoS, Beacon flood, Authentication flood	Availability	In a complex Smart Healthcare system, only authorized users and perhaps other systems should be able to access wireless network resources anytime and anywhere upon request.	Techniques such as spread spectrum techniques, direct-sequence spread spectrum, frequency-hopping spread spectrum can be employed [66] to mitigate such threats for IoT medical devices.
Impersonation, Password, Dictionary, Brute-force, Sniffer, Spoofing, Access aggregation	Authenticity	Specified to differentiate authorized users from unauthorized users. In Smart Healthcare systems authentication is crucial for all participating entities (patients, medical staff point, devices, etc.)	Use medium access control (MAC) address for authentication purposes. Also use network-layer authentication, transport-layer authentication and application layer authentication [66].

## Abbreviations

The following abbreviations are used in this manuscript:

API	Application Programming Interface
BAN	Body Area Network
CoAP	Constrained Application Protocol
COTS	Commercial Off-The-Shelf
CP-ABE	Ciphertext-Policy Attribute-Based Encryption
CSMA/CA	Carrier Sense Multiple Access/Collision Avoidance
DBPSK	Differential Binary Phase-Shift Keying
DL	Downlink
DSSS	Direct Sequence Spread Spectrum
EC-GSM-IoT	Extended Coverage – GSM – Internet of Things
ECG	Electrocardiogram
EEG	Electroencephalogram
EGPRS	Enhanced General Packet Radio Service
EHR	Electronic Health Records
EMG	Electromyogram
FDA	Food and Drug Administration
FDMA	Frequency Division Multiple Access
FHSS	Frequency Hopping Spread Spectrum
FSK	Frequency Shift Keying
GFSK	Gaussian Frequency Shift Keying
HBC	Human Body Communications
IoT	Internet of Things
IoMT	Internet of Medical Things
IMD	Implantable Medical Devices
ISM	Industrial, Scientific and Medical
LECIM	Low-Energy Critical Infrastructure Monitoring
LLN	Low-power and Lossy Networks
LDPC	Low Density Parity Check
LoRaWAN	Long Range Wider Area Network
LP-WAN	Low Power Wide Area Network
LR-WPAN	Low Rate - Wireless Personal Area Network
LTE CAT-N	Long Term Evolution Category N
LTE-M	Long Term Evolution - Machine Type Communications
MAC	Medium Access Control
MITM	Man-In-The-Middle
MCS	Mobile Crowdsensing
MTC	Machine Type Communications
NB-IoT	Narrow Band Internet of Things
NFC	Near Field Communication
OFDM	Orthogonal Frequency Division Multiplexing
OTA	Over-the-Air
PER	Packet Error Rate
PSK	Phase Shift Keying
RAW	Restricted Access Window
QC	Quantum Computing
QKD	Quantum Key Distribution
QoI	Quality of Information
QoS	Quality of Service
QPSK	Quadrature Phase shift Keying
QRNG	Quantum Random Number Generator
REST	REpresentational State Transfer
RFD	Radio Frequency
RFD	Radio Frequency Identification
PASH	Privacy-Aware Smart Health
RHM	Remote Health Monitoring
RPMA	Random Phase Multiple Access
RPL	Routing Protocol for Low-Power and Lossy Networks
SDN	Software-Defined Networking
SHR	Smart Health Record
SpO2	Oxygen Saturation
SRD	Short Range Device
TDMA	Time Division Multiple Access
TSCH	Time-Slotted Channel Hopping
UL	Uplink
UWB	Ultra-WideBand
WBAN	Wireless Body Area Network
WMDs	Wearable Medical Devices
WSN	Wireless Sensor Network
6LoWPAN	IPv6 over Low - Power Wireless Personal Area Networks
6TiSCH	IPv6 over the TSCH
